# Rectal impalement injury through the pelvis, abdomen and thorax

**DOI:** 10.1308/003588412X13373405385016

**Published:** 2012-09

**Authors:** LC Ho, H El Shafei, J Barr, B Al Kari, EH Aly

**Affiliations:** ^1^University of Aberdeen,UK; ^2^NHS Grampian,UK

**Keywords:** Rectal impalement injury, Penetrating chest-abdomen-pelvis injury

## Abstract

Impalement rectal injuries with intraperitoneal organ injuries are rare. It is even rarer for such injuries to result in pelvic, abdominal and thoracic internal injuries. We present the case of a 39-year-old man who was admitted after an assault where a broken broomstick was inserted forcibly into his rectum. Surgery revealed penetration through the rectum, dome of the bladder, mesentery, liver and right lung. The patient survived following management by a multispecialty surgical team. Our literature review identified four similar cases with one fatality only. Prognosis seems to be good in these types of injuries provided there is an early presentation, the penetrating object is left in situ before the operation and, most importantly, an organised team approach to deal with the various injuries.

Rectal injuries are rare as they only represent 9.6% of all colorectal trauma. In most series, gunshot wounds account for 80–85% of injuries and stab wounds for 3–5%. Other causes include iatrogenic injuries from urological and endoscopic procedures, sexual misadventures and anorectal foreign bodies. Blunt trauma accounts for 5–10% of cases and is usually the result of pelvic fractures or impalement.

One of the earliest attempts to review these injuries was in 1896 and included 58 cases.^2^ Rectal injuries that penetrate the peritoneum are even less common as in a subsequent more comprehensive review of 175 cases, peritoneal penetration was recorded in only 70 cases. Rectal impalement injuries with penetrating trauma across the pelvis, abdomen and thorax are much rarer. We report a case of such an injury, where the patient presented after an assault and survived with no major lasting physical damage.

## Case history

A 39-year-old man presented as an emergency following assault where a wooden handle of a cleaning mop had been forcibly inserted into his rectum while his head was slammed in a car door. The mechanism of the injury was low velocity as he had been bent over and one of the assailants pushed the wooden handle in via his foot.

On examination, there were abrasions and bruises periorbitally, behind his ear and around his trunk. There was also bleeding per rectum. Nevertheless, he was haemodynamically stable with a Glasgow coma scale score of 15.

**Figure 1:**
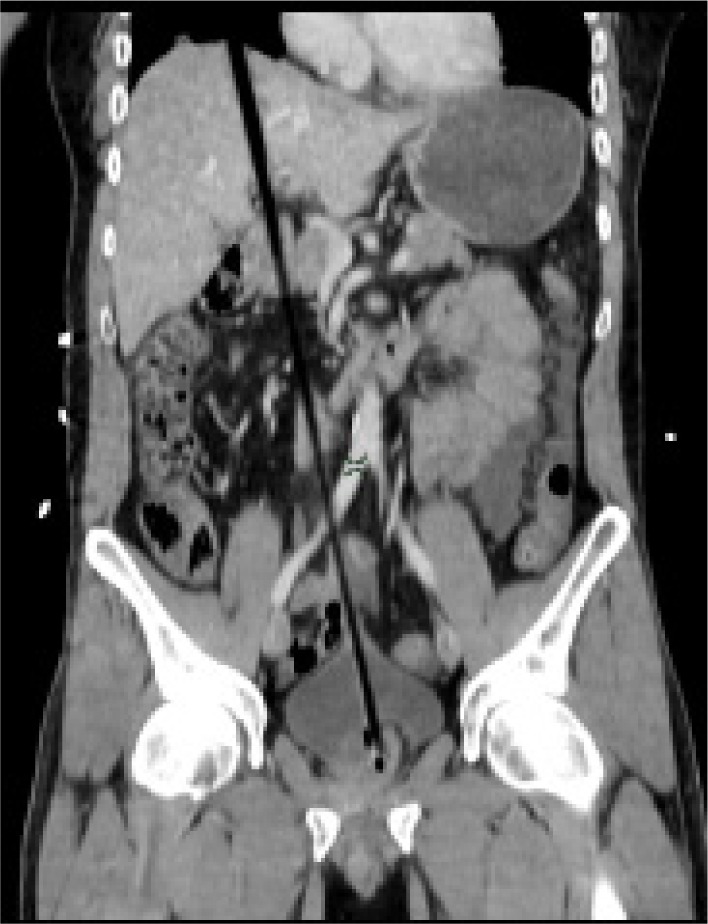
Coronal computed tomography of abdomen and pelvis showing extent of impalement

**Figure 2:**
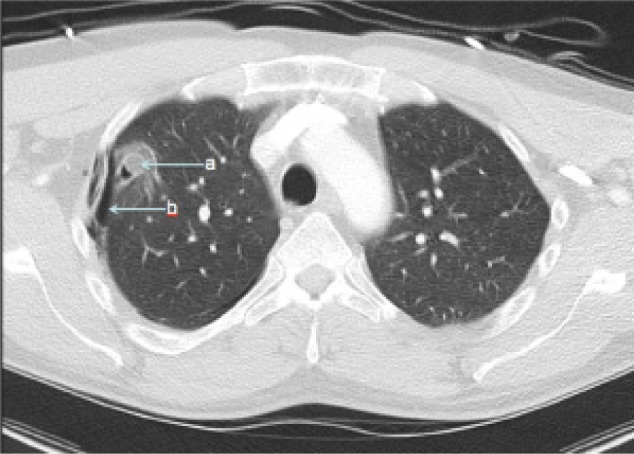
Computed tomography of thorax showing: a) stick and b) pneumothorax

**Figure 3:**
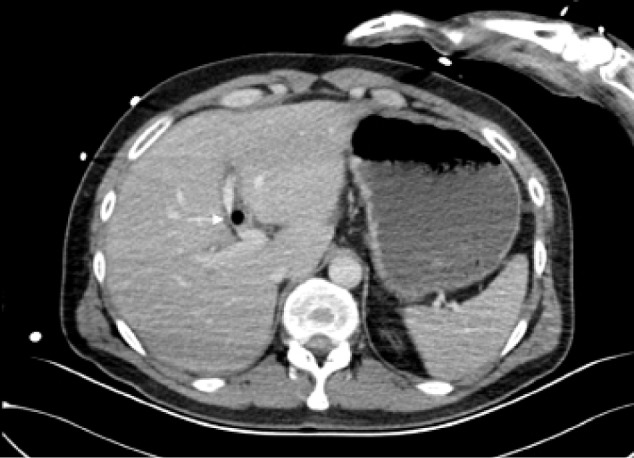
Computed tomography of abdomen showing the stick traversing the liver

Computed tomography (CT) of the head and spine ruled out head and spinal injuries. However, CT of the chest, abdomen and pelvis revealed a foreign body penetrating from the lower third of the rectum through the bladder, mesentery, stomach and liver, and to the right lung (Figs 1 – 3). A small right pneumothorax was noted (Fig 2). The patient was taken to theatre and a chest drain was put in. This was followed by an emergency laparotomy and thoracotomy, a joint procedure involving three consultant surgeons in the fields of colorectal, cardiothoracic and hepatopancreatobiliary surgery.

Examination under anaesthesia did not reveal a palpable mucosal defect in the rectum. Following laparotomy, a wooden rod was seen to penetrate the rectum, dome of the bladder, root of the small bowel mesentery, transverse mesocolon, anterior and posterior walls of the stomach, inferior to superior surface of the liver through the porta hepatis, diaphragm and right lung. The upper tip of the wooden stick was lying in contact with the right subclavian vessels. However, there was no associated vascular injury.

The wooden rod was divided in its middle below the liver using an electric saw and removed from the root of the mesentery and a Hartmann’s operation with an end colostomy was performed. The liver was partially mobilised with hilar control and the upper part of the rod was removed slowly under direct vision. There was some bile leak from the exit wound in the superior aspect of the liver. A drain was left in and a decision was made that if the leak continued, a post-operative endoscopic retrograde cholangiopancreatography (ERCP) would be considered. Lacerations in the stomach and dome of the bladder were repaired. The thoracotomy revealed 1–2 litres of blood clots and haemostasis was achieved.

The patient was transferred to the intensive care unit. He was successfully weaned off ventilatory support and discharged to the surgical high dependency unit after one day. He had post-operative bile leak that stopped after few days and ERCP was therefore not needed.

A Gastrografin® enema (Bracco Diagnostics, Princeton, NJ, US) ten weeks later revealed no leak from the rectal stump and a month later the patient underwent an elective laparoscopic reversal of his Hartmann’s operation. Post-operative recovery was uneventful.

## Discussion

There has been progressive decline in the mortality from colorectal injuries.^3^ The mortality rate decreased to 40% during the Second World War from more than 90% during the US Civil War (1861–1865) and subsequently to less than 10% through the Vietnam War. Even though there has been marked improvement in the management of colorectal injuries, the mortality rate is still 3% and the abdominal sepsis rate is higher than 20%.

There have been only four previously reported similar cases in the literature of rectal impalement injury though the pelvis, abdomen and thorax (Table 1). Our case highlights the advantage of a multispecialty approach.

**Table 1:** Summary of the previous reports of rectal impalement injury though the pelvis, abdomen and thorax

Authors	Age and sex	Mechanism of injury	Object	Associated injuries	Complications	Outcome
Boyd^2^	16M	Accidental fall during play	Metal crossbar	Left external iliac and anterior rectal wall lacerations	Rectourethral fistula; loss of left kidney function as left ureter fibrosed due to infection	Survived
Lear *et al*^4^	26F	Jump from burning building	Tree branch	Devascularised right colon and terminal ileum; lacerations in right lobe of liver and broad ligament	Bilateral bronchopneumonia; paralytic ileus septicaemia; required psychiatric treatment for post-traumatic effects	Survived
Karger *et al*^5^	49M	Possibly self inflicted	Stool leg	Intra-abdominal haemorrhage; lacerations in anterior rectum, bladder, peritoneum, mesentery, transverse mesocolon and liver	Died
Moncure *et al*^6^	45M	Possibly self-inflicted	Broomstick	Lacerations in mesentery, stomach, diaphragm and pericardium	Atrial fibrillation; pneumonia; brachial plexopathy; ventral incisional hernia; residual right upper limb weakness	Survived

Rectal impairment injuries that also involve the abdominal and thoracic cavities are usually due to accidents,^2,4^ sexually related activities^5,6^ or, as in our case, assault. The objects used for penetration tend to be long and thin such as mop handles, chair legs, tree branches or billiard cues.^2^ Diagnosis is often challenging; in our patient’s case there was no visible perineal trauma and even a rectal examination under anaesthesia revealed no mucosal defect. Clinical examination only is therefore not reliable to diagnose rectal injuries, their extent and associated organ damage.^7^ CT examination allowed us to establish the diagnosis and also to assess pre-operatively the extent of the various injuries, which in our case was far greater than could be predicted from clinical examination only. More importantly, it allowed planning of the surgical intervention before the start of the operation as well as coordination between different specialties.

Despite our patient’s injury involving the pelvic, abdominal and thoracic cavities, it was not associated with major vascular injury. This has also been commented on by other authors.^6^ This could be explained by the fact that in both occasions the foreign object had a rounded end, acting as a blunt tunneler. As the foreign object in our case was pushed by the assailant’s foot, it travelled with low velocity, resulting in displacement rather than penetration of the major vascular structures, especially in the porta hepatis.

## Conclusions

Prognosis seems to be good in these types of injuries provided there is an early presentation, the penetrating object is left in situ before the operation and, most importantly, there is an organised team approach to deal with the various injuries.
